# Fault Diagnosis Method for Human Coexistence Robots Based on Convolutional Neural Networks Using Time-Series Data Generation and Image Encoding

**DOI:** 10.3390/s23249753

**Published:** 2023-12-11

**Authors:** Seung-Hwan Choi, Jun-Kyu Park, Dawn An, Chang-Hyun Kim, Gunseok Park, Inho Lee, Suwoong Lee

**Affiliations:** 1 Advanced Mechatronics Research Group, Daegyeong Division, Korea Institute of Industrial Technology, Daegu 42994, Republic of Korea; csw1496@kitech.re.kr (S.-H.C.); limition@kitech.re.kr (C.-H.K.); rjstjr010@kitech.re.kr (G.P.); 2 Renewable Energy Solution Group, Korea Electric Power Research Institute (KEPRI), Naju 58277, Republic of Korea; junq14@kepco.co.kr; 3 Department of Electronics Engineering, Pusan National University, Busan 46241, Republic of Korea

**Keywords:** human coexistence robots, fault diagnosis, Wasserstein generative adversarial networks with gradient penalty, image encoding, recurrence plot, Gramian angular field, Markov transition field, spectrogram, scalogram, convolutional neural network

## Abstract

This paper proposes fault diagnosis methods aimed at proactively preventing potential safety issues in robot systems, particularly human coexistence robots (HCRs) used in industrial environments. The data were collected from durability tests of the driving module for HCRs, gathering time-series vibration data until the module failed. In this study, to apply classification methods in the absence of post-failure data, the initial 50% of the collected data were designated as the normal section, and the data from the 10 h immediately preceding the failure were selected as the fault section. To generate additional data for the limited fault dataset, the Wasserstein generative adversarial networks with gradient penalty (WGAN-GP) model was utilized and residual connections were added to the generator to maintain the basic structure while preventing the loss of key features of the data. Considering that the performance of image encoding techniques varies depending on the dataset type, this study applied and compared five image encoding methods and four CNN models to facilitate the selection of the most suitable algorithm. The time-series data were converted into image data using image encoding techniques including recurrence plot, Gramian angular field, Markov transition field, spectrogram, and scalogram. These images were then applied to CNN models, including VGGNet, GoogleNet, ResNet, and DenseNet, to calculate the accuracy of fault diagnosis and compare the performance of each model. The experimental results demonstrated significant improvements in diagnostic accuracy when employing the WGAN-GP model to generate fault data, and among the image encoding techniques and convolutional neural network models, spectrogram and DenseNet exhibited superior performance, respectively.

## 1. Introduction

The active adoption of human coexistence robots (HCRs), such as collaborative robots and autonomous mobile robots, in industrial settings has recently led to significant improvements in productivity and efficiency. These robots have inherent capabilities that enable them to independently carry out simple and repetitive tasks in industrial environments. However, the true value of HCR lies in their ability to collaborate with humans in shared spaces. This marks a new era in robot technology and highlights the central role these robots will play in future industrial landscapes [1,2].

Given that HCRs work closely alongside humans in the same environment and perform a variety of tasks, ensuring stable and precise management is essential. Any malfunction or incorrect operation during a task could pose a serious risk to workers. Consequently, the stable operation of HCRs has become a critical issue, directly impacting both work productivity and worker safety [3,4,5].

Current research trends are primarily focused on real-time monitoring of the operational status of HCR, as well as the early detection of abnormal behaviors or potential faults. To achieve this, a combination of various sensor technologies, AI-based diagnostic methods, and machine learning strategies for prognostics and health management is being employed. Sensors monitor various operational parameters of the robot, and AI-based algorithms analyze this data to identify anomalies or potential issues in the robot’s operation [2,5,6].

The majority of faults in HCRs occur in the core component known as the driving module. This module consists of elements such as gear reducers, bearings, motors, encoders, and brakes, and it controls the robot’s movements through rotation. Robots are utilized in diverse working environments within industrial settings, each with varying working times and methods. Additionally, the types of faults in the driving module can vary, and the data characteristics associated with each fault are often difficult to distinguish. Therefore, even if robots have the same specifications and perform identical tasks in the same space, the types and characteristics of faults may differ. Hence, ongoing research is primarily directed at diagnosing faults in rotating components like the driving module [7,8].

Traditionally, the process of diagnosing faults in robots and rotating machinery in-volved extracting features from raw time-series data through preprocessing techniques like normalization, moving average, differencing, Fourier transform, and wavelet trans-form. These features were then used for training and classification within neural network models. However, dealing with diverse features and detecting faults using 1D data proved to be a challenge. Recently, research has increasingly shifted towards converting 1D time-series data into 2D images for feature extraction and fault detection, and advancements in deep learning have already demonstrated excellent performance in image classification [3,9,10,11].

The advantages of fault diagnosis using 2D Images are as follows:

- Fault diagnosis based on 2D images does not require the need for complex feature extraction processes, predefined parameters, and expert knowledge required in traditional 1D data-based diagnostic methods, thereby shortening experimental procedures and time [12,13].
- Two-dimensional images inherently contain more information than 1D data and possess visual characteristics, allowing for more diverse feature extraction and effective fault diagnosis when applied to CNN models [14,15].
- Two-dimensional image-based fault detection enables quick and easy identification of various data characteristics without direct feature extraction, offering low time complexity and being well-suited for real-time, high-precision fault diagnosis [12,16].

As image recognition technology continues to advance, ongoing efforts aim to apply similar technologies to time-series data. Recent research has compared traditional 1D time-series data approaches with image encoding techniques, presenting findings that substantiate the superior efficacy of image encoding methods [17,18]. Representative image encoding techniques include recurrence plot (RP) [19,20,21,22,23], Gramian angular field (GAF) [14,24,25,26,27], Markov transition field (MTF) [28,29,30], spectrogram (SP) [31,32], and scalogram (SC) [33,34]. These image encoding techniques have recently been applied in research that converts time-series data from vibration and current signals, collected for diagnosing faults in robots and various machinery (such as bearings, gearboxes, rotating machinery, complex distribution networks, ventilation, and air conditioning systems), into images for various convolutional neural network (CNN) models.

In real industrial environments, the persistent challenges of data scarcity and diversity stem from varying environmental conditions and the mechanical characteristics of robots used for fault diagnosis. Moreover, in practical industrial settings, robots and machinery often cease operations when a fault occurs, making it extremely challenging to obtain data regarding the fault state. Consequently, the field of data generation technology has been explored, with methods like variational autoencoders (VAE), generative adversarial networks (GAN), and diffusion models being proposed. Among these, the most prominent data generation model is GAN. The core structure of a GAN model consists of two networks: a generator, which generates synthetic data from random noise, and a discriminator, which distinguishes between real and synthetic data. These two networks en-gage in competitive learning, thereby enhancing the model’s performance over time. Through this process, the generator progressively creates more sophisticated fake data, while the discriminator becomes more adept at accurately discerning real data from syn-thetic data [35,36,37].

The overall contribution of the proposed work can be summarized as follows:

1. Time-series vibration data obtained from the durability tests of drive modules for HCR are divided into sections representing normal and faulty conditions considering lifespan and stability, and data samples are extracted.
2. To compensate for the significantly lower amount of fault data compared to normal data, additional samples are generated using the Wasserstein GAN with gradient penalty (WGAN-GP) model.
3. The 1D data for both normal and fault states are arranged in a specified sequence and then applied to five image encoding techniques (recurrence plot, Gramian angular field, Markov transition field, spectrogram, and scalogram), converting them into corresponding image data.
4. The image data are applied to four CNN models (VGGNet, GoogleNet, ResNet, and DenseNet) to calculate the accuracy of fault diagnosis and provide the most suitable algorithm for the dataset by comparing the performance of each model.

The remainder of this paper is organized as follows: Section 2 describes the durability test methods of the driving module for data collection, Section 3 introduces the data generation and image encoding techniques proposed in this study. Section 4 calculates the accuracy of fault diagnosis using image encoding and CNN models, and compares the performance of each algorithm. Finally, Section 5 provides the conclusions of this paper.

## 2. Durability Test Method for Driving Module of HCR

**Figure 1:**
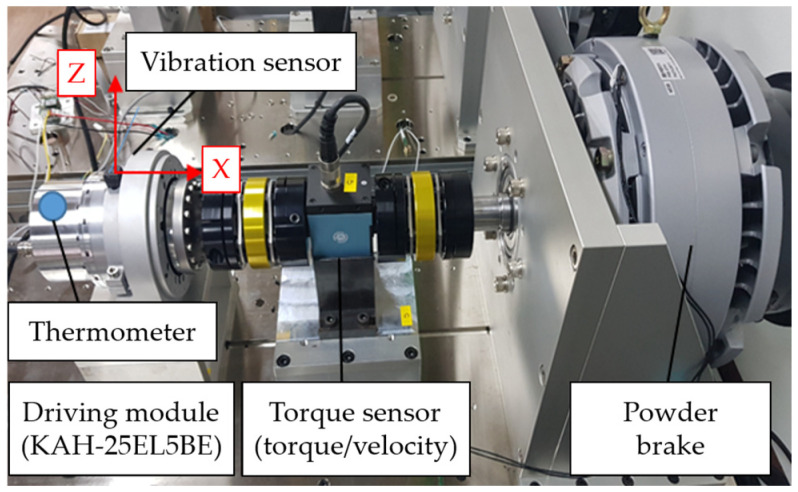
Durability test environment for the driving module of HCR.

Figure 1 depicts the test environment for assessing the durability of the driving module used in HCR. The specific driving module utilized is the KaiserDrive KAH-25EL 5BE model, characterized by a rated power of 500 W, a reduction ratio of 160, a maximum average torque of 133 Nm, and a rated torque and speed of 84 Nm and 12 RPM, respectively. The vibration sensor employed is the PCB 356A17 model, which is a three-axis accelerometer with a sensitivity of 500 mV/g, a measurement range of ±98 m/s^2^ pk, and a frequency range spanning from 0.4 to 4000 Hz. In the course of the durability test, speed and torque settings were chosen in accordance with the specifications of the driving module and a series of repeated tests were carried out by regulating a powder brake to execute the defined operations.

The driving conditions for the durability test of the driving module are summarized in Table 1. The module was continuously operated until faulty, repeatedly rotating in both forward and reverse directions according to the set torque and speed patterns.

**Figure 2:**
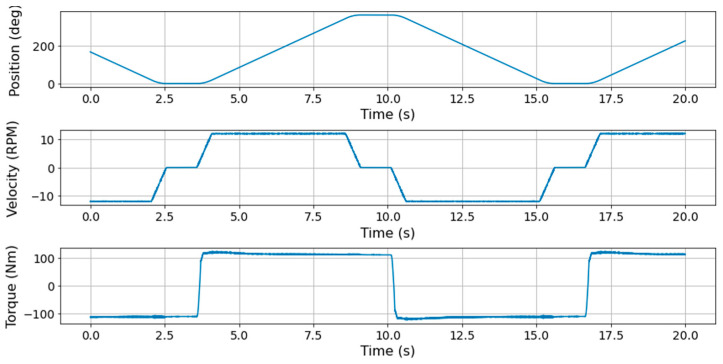
Position, speed, and torque patterns in the durability test of the driving module.

Figure 2 illustrates the procedure for conducting the durability test on the driving module. The driving module underwent a cyclic forward/backward rotation spanning angles from 0° to 360°. This cycle included the following sequence: acceleration (0.5 s) → constant speed (4.5 s) → deceleration (0.5 s) → stop (1 s). Data were captured through a vibration sensor at a sampling rate of 1 kHz, with 20 s of data collected every 10 min. Given that vibration characteristics vary with rotation speed, only the data from the constant-speed phase during forward rotation was extracted and utilized in the fault diagnosis model. Furthermore, recognizing that the number of constant-speed data points can differ based on the operation pattern within each cycle, only 5 s of constant-speed data were extracted per cycle and employed in the fault diagnosis model.

**Figure 3:**
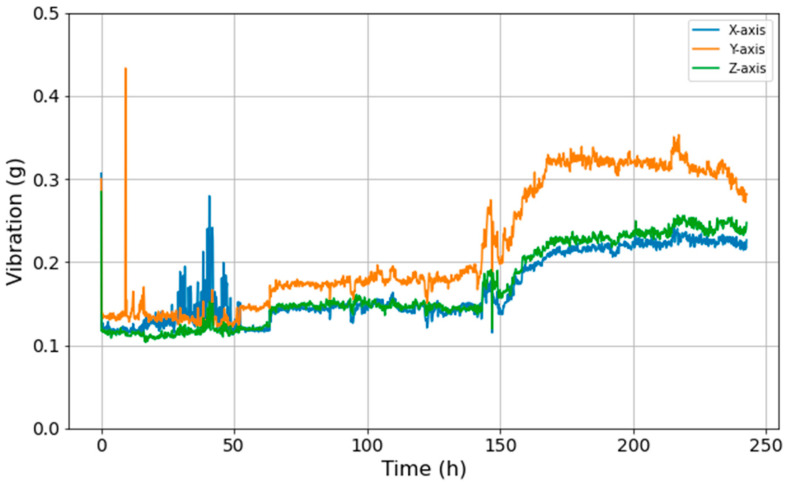
Time-series vibration data based on the durability test of the driving module.

Figure 3 demonstrates the magnitude of vibrations observed during the durability test of the driving module. Initially, the driving module displayed pronounced vibrations that subsequently reached a stable state. Notably, the magnitude of vibrations saw an uptick around the 150 h mark, with a fault occurring at approximately 243 h.

During the durability test, the driving module stopped functioning due to a fault, and no data were obtained after the fault occurrence. Nevertheless, in actual industrial settings, the pre-emptive diagnosis of faults using data immediately prior to the fault is of greater importance than utilizing post-fault data, taking into account factors like productivity and maintenance efficiency. Therefore, this study opted to utilize data from the moments just before the fault as fault data and designed a model trained to diagnose faults using both normal and fault data.

## 3. Techniques of Data Generation and Image Encoding

### 3.1. Data Generation Mothod

#### 3.1.1. Time-Series Data Generation Model

**Figure 4:**
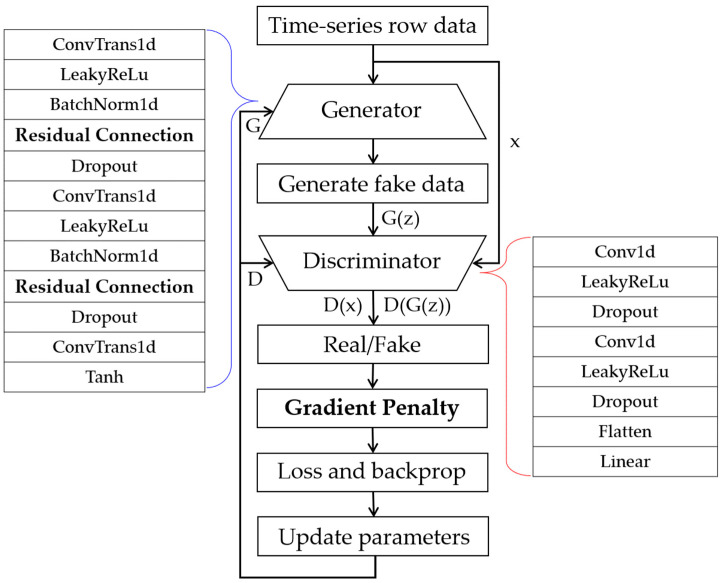
Structure of Wasserstein generative adversarial networks with gradient penalty (WGAN-GP) for data generation.

In this study, an enhanced version of the Wasserstein GAN (WGAN), known as WGAN-GP, was employed to generate the limited fault data. Figure 4 displays the architecture of the WGAN-GP utilized in this study. To preserve essential features in the input data and enhance the learning capability of the model while retaining its fundamental structure, residual connections were introduced to the generator. Furthermore, to mitigate training instability often linked to mode collapse in traditional GANs, we enhanced the training process by incorporating the Wasserstein distance. This addition, along with the introduction of a gradient penalty, addresses the issues of gradient vanishing/exploding in the discriminator, thus promoting more effective learning [38,39,40,41,42].

#### 3.1.2. Similarity Evaluation Method of Generated Data

The objective of the data generation model is to produce data that closely resemble real data. Therefore, a method is required to assess the degree of similarity between the generated synthetic data and real data. In this study, we employed statistical parameters, including mean, median, standard deviation (SD), skewness, and kurtosis, to evaluate and compare the similarity between the generated and fault data. Greater similarity in the values of these statistical attributes between the generated and fault data signifies more effective data generation.

**Figure 5:**
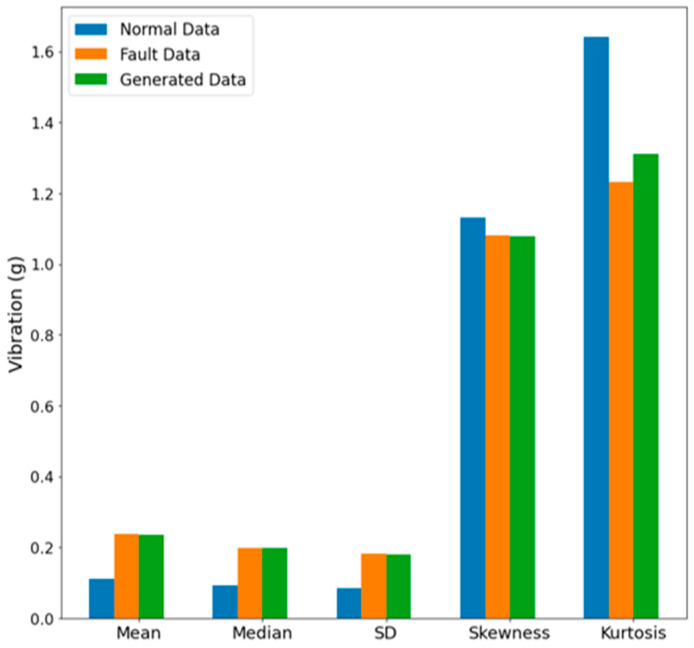
Comparison of statistical properties of real and generated data.

Figure 5 illustrates a graph comparing the statistical attribute values of normal, fault, and generated data, with the actual values listed in Table 2. The generated data were observed to exhibit a close resemblance to the fault data in all statistical aspects, including mean, median, SD, skewness, and kurtosis, when compared to the normal data.

### 3.2. Image Encoding Methods from Time-Series Data

Several methods have been proposed for transforming the 1D time-series data into 2D images. Recognized image encoding techniques comprise RP, GAF, MTF, SP, and SC.

#### 3.2.1. Image Encoding Methods

RP is an image visualization method designed to examine m-dimensional phase space trajectories by illustrating the recurrence of data values in 2D [16,17]. When time-series data are provided as $X=\left\{x_{1},\;x_{2},\;\cdots ,\;x_{i}\right\}$, the m-dimensional phase space trajectory S can be expressed as Equation (1).

(1) $$S=\{s_{1}=\left(x_{1},\;x_{2},\right),{\;s}_{2}=\left(x_{2},\;x_{3},\right),\;\cdots ,\;s_{n}=\left(x_{n},\;x_{n+1}\right)\}$$

where $s_{n}$ is the trajectory of time-series data from $x_{n}$ to $x_{n+1}$. Upon establishing the m-dimensional phase space trajectory of the time-series data, the Euclidean distances between these trajectories are illustrated in a matrix. This comprehensive matrix of distances is referred to as the RP matrix, denoted by Equation (2).

(2) $$R_{i,j}=\Theta \left(\epsilon -∥\vec{s_{l}}-\vec{s_{j}}∥\right)$$

where $\Theta \left(x\right)$ is the Heaviside function, which outputs 1 if the value of x is greater than or equal to 0 and outputs 0 if it is less than 0; ϵ represents the threshold value. RP employs trajectory recurrence to offer insights into the periodicity and amplitude variations within time-series data, effectively extracting feature information by visually depicting the data’s volatility and correlations in an image.

GAF is a method that visualizes the temporal correlation between various time points in time-series data using polar coordinates [22,23]. When the time-series data are denoted as $X=\left\{x_{1},\;x_{2},\;\cdots ,\;x_{i}\right\}$, the Gram matrix (G) can be expressed as follows:

(3) $$G=\left\{\begin{array}{ccc} \left(x_{1},x_{1}\right) & \ldots & \left(x_{1},x_{n}\right)\\ \vdots & \ddots & \vdots \\ \left(x_{n},x_{1}\right) & \ldots & \left(x_{n},x_{n}\right) \end{array}\;\right\}$$

GAF normalizes the time-series data X to the range [−1, 1] or [0, 1] and transforms the data into polar coordinates as it changes over time.

(4) $$\left\{\begin{array}{c} \phi =arccos\left(\tilde{x_{i}}\right),-1\leq \tilde{x_{i}}\leq 1,\;and\;\tilde{x_{i}}\in \tilde{X}\\ r_{i}=\frac{t_{i}}{N},\;t_{i}\in N \end{array}\right.$$

The normalized time-series data $\tilde{x_{i}}$ is transformed into the arccosine function using Equation (4), and the time $t_{i}$ is calculated as the radius $r_{i}$ to transform it into polar coordinates. Subsequently, a matrix is generated by computing the dot product between each data point and is portrayed as an image. Here, N represents a constant used to normalize the polar coordinate range.

GAF is presented in two variations, based on the summation and difference of angles. GASF symbolizes the summation of angles in polar coordinate time-series data, involving time pairs i and j, and is defined by Equation (5).

(5) $$\mathrm{GASF}=\left[\cos\left(\phi_{i}+\phi_{j}\right)\right]={\tilde{x}}^{\prime }\cdot \tilde{x}-{\sqrt{I-{\tilde{x}}^{2}}}^{\prime }\cdot \sqrt{I-{\tilde{x}}^{2}}$$

Conversely, GADF is defined by the difference in angles of the polar coordinates, as follows:

(6) $$\mathrm{GADF}=\left[\sin\left(\phi_{i}-\phi_{j}\right)\right]={\sqrt{I-{\tilde{x}}^{2}}}^{\prime }\cdot \tilde{x}-{\tilde{x}}^{\prime }\cdot \sqrt{I-{\tilde{x}}^{2}}$$

GADF transforms into a 2D matrix using the sine function for the difference as given in Equation (6), while GASF employs the cosine function for the summation as described in Equation (5). By means of dot products, time-series data with temporal attributes are converted into a matrix. As one traverses from the top-left to the bottom-right of the matrix, the temporal progression is maintained, and GAF conserves visual relationships through the matrix’s geometry.

MTF is a technique that converts discretized time-series data into images based on transition probabilities [25,26,27]. This approach captures the dynamic characteristics of state transitions within the time-series data and visualizes them in the form of a 2D image.

When provided with time-series data $\left\{x_{1},\;x_{2},\;\cdots ,\;x_{i}\right\}$, MTF initially discretizes the data into multiple states. It then computes the transition probabilities between each state to create a Markov chain. The resulting transition probability matrix can subsequently be converted back into an image.

MTF organizes each probability in chronological order and defines it as shown in Equation (7). Here, $w_{ij}$ represents the frequency of transitioning from the $q_{i}$ interval to the $q_{j}$ interval. The value $M_{ij}$ in the ith row and jth column of M represents the probability of transitioning from the interval $q_{i}$ containing the data value at time index $t_{i}$ to the interval $q_{j}$ containing the data value at time index $t_{j}$. If the interval width is large, most values are aggregated in the interval closest to the mean, and if the interval width is small, fewer values are aggregated in the extreme intervals.


(7) $$M_{ij}=\left[\begin{array}{cccc} M_{11} & M_{12} & \cdots & M_{1n}\\ M_{21} & M_{22} & \cdots & M_{2n}\\ \vdots & \vdots & \ddots & \vdots \\ M_{n1} & M_{n2} & \cdots & M_{nn} \end{array}\right]=\left[\begin{array}{ccc} w_{ij}|x_{1}\in q_{i},x_{1}\in q_{j} & \cdots & w_{ij}|x_{1}\in q_{i},x_{n}\in q_{j}\\ w_{ij}|x_{2}\in q_{i},x_{1}\in q_{j} & \cdots & w_{ij}|x_{2}\in q_{i},x_{n}\in q_{j}\\ \vdots & \ddots & \vdots \\ w_{ij}|x_{n}\in q_{i},x_{1}\in q_{j} & \cdots & w_{ij}|x_{n}\in q_{i},x_{n}\in q_{j} \end{array}\right]$$

The images produced by the MTF technique provide a visual representation of Markov transition dynamics, revealing patterns or periodicities within the time-series data in diverse ways. Consequently, these images prove valuable for pattern recognition or comparative analysis, which can be challenging when dealing with the original time-series data.

SP is a method for illustrating changes in the frequency domain over time in time-series data. SP generates a 2D image by employing time and frequency as the two axes and employs color to represent energy density at their intersections [28,29]. The widely used algorithm is the short-time Fourier transform (STFT), which divides the time-series data into small sections of a specific size and calculates the Fourier transform for each section. These outcomes offer insight into the variations in energy density across different frequency components over time.

(8) $$SP\left(t,f\right)={\left|STFT\left(x\left(t\right),\;f\right)\right|}^{2}$$

where $x\left(t\right)$ denotes the time-series data, f denotes frequency, and $SP\left(t,f\right)$ denotes the energy density at time t and frequency f.

SC is a type of wavelet transform that presents changes in frequency components over time in time-series data as a 2D image [30,31]. Similar to SP, SC also employs time and scale (inversely related to frequency) as its two axes, using color to depict energy density at specific locations.

(9) $$SC\left(t,s\right)={\left|WT\left(x\left(t\right),s\right)\right|}^{2}$$

where WT represents the wavelet transform, $x\left(t\right)$ represents the time-series data, and s represents scale.

SC is particularly effective for analyzing complex and nonlinear patterns in non-stationary time-series data, particularly in detecting energy changes across various frequency bands.

#### 3.2.2. Image Encoding of Time-Series Data

**Figure 6:**
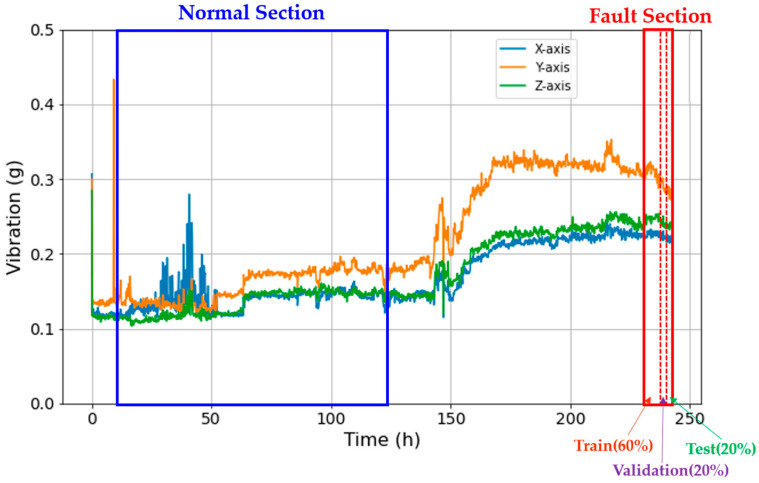
Data extraction from normal and fault sections in all vibration data.

In this study, data were collected up to the point just before failure occurred, using durability tests of the driving module. Given the absence of data obtained after a failure, to apply a classification-based fault diagnosis model, data sections for both normal and fault conditions were designated as depicted in Figure 6, with 10 h of data extracted from each section. Considering the characteristic changes due to the aging of the modules and the stability of the data, the normal section was selected as the initial 50% of the module’s total lifespan, excluding the first 10 h, and the fault section as the last 10 h before the failure. An equivalent number of samples were then collected from each section. Furthermore, considering the continuity of the time-series data, we avoided random shuffling of the data for training, validation, and test sets. Additionally, in consideration of the continuity of time-series data, random mixing of data for training, validation, and testing was avoided. Instead, as illustrated in Figure 6, datasets were divided sequentially starting from the earlier data, allowing the test set to incorporate data that closely approximates actual fault conditions.

**Figure 7:**
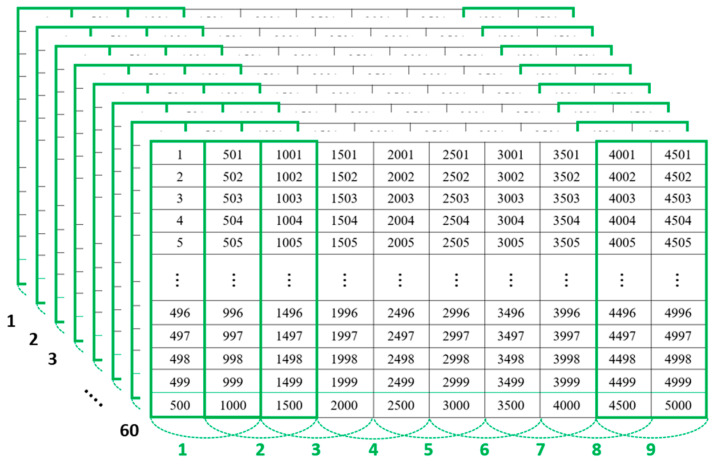
Method for data segmentation for image creation based on image encoding.

Data for normal and fault conditions were extracted every 10 min, selecting only 5000 points from the constant-speed sections. The collected data were then transformed into images, following the procedure detailed in Figure 7, resulting in a total of 9 images from 5000 data points. For the creation of a single image, 1000 data points were utilized. Additionally, to increase the number of images, an overlap of 50% of the data was employed. Therefore, 540 images were created each from the data of normal and fault conditions, both based on a 10 h timeframe.

**Figure 8:**
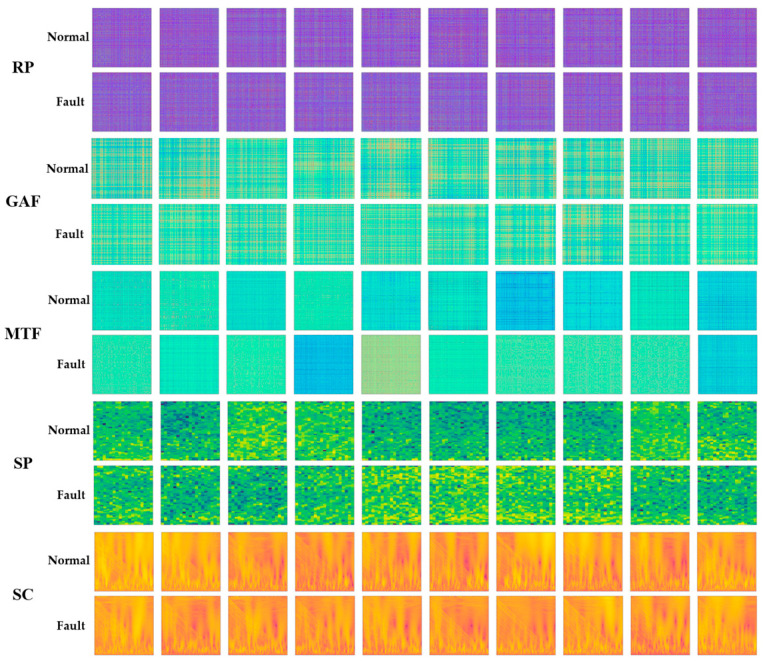
Recurrence plot (RP), Gramian angular field (GAF), Markov transition field (MTF), spectrogram (SP), and scalogram (SC) images transformed through image encoding techniques.

Figure 8 presents the images of normal and fault conditions that were transformed using the image encoding techniques of RP, GAF, MTF, SP, and SC. Various images with different characteristics were generated according to each technique, and visually, it is difficult to distinguish between images of normal and fault conditions. Therefore, in this study, image data were generated using five different image encoding techniques, and the performance of fault diagnosis was evaluated by utilizing CNN models to effectively extract image features.

## 4. Results and Discussion

This study proposes a method for fault diagnosis using limited fault data, image encoding techniques, and a CNN model. The experiments were divided into two parts: fault diagnosis utilizing image encoding techniques and a CNN model, and fault diagnosis subsequent to generating fault data and applying image encoding techniques and a CNN model. The performance of these approaches was subsequently compared.

### 4.1. Fault Diagnosis Using Image Encoding and CNN Models

**Figure 9:**
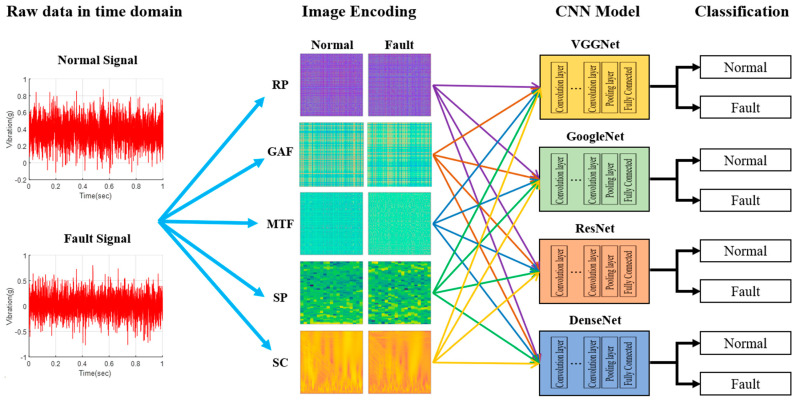
Method of fault diagnosis using image encoding techniques and convolutional neural network (CNN).

Figure 9 illustrates the process of fault diagnosis using image encoding techniques and a CNN model. This research utilized the CNN models VGGNet, GoogleNet, ResNet, and DenseNet, all proven to be effective in image recognition and classification. Time-series data of normal and fault conditions were converted into images using five types of image encoding techniques. These images were then applied to four types of CNN models to calculate the accuracy of fault detection for each. Furthermore, the same model was subjected to 10 tests, and the average was computed to determine the final accuracy.

In this study, the algorithm was implemented on a PC with the following specifications: CPU: Intel(R) Core(TM) i7−12700 at 2.10 GHz, GPU: NVIDIA GeForce RTX 3080, RAM: 128 GB. The algorithm was implemented in a Python environment. The computing time for converting 10 h of time-series data into 540 images using image encoding techniques was as follows: RP took 197.94 s, GAF 222.44 s, MTF 197.51 s, SP 27.01 s, and SC 77.43 s. For the computing time for the fault diagnosis process through CNN models, based on Epoch 20, the times were: VGGNet 168.12 s, GoogleNet 203.45 s, ResNet 180.64 s, and DenseNet 358.34 s. The total computing time for implementing the algorithm is as shown in Table 3. It was observed that in terms of computing speed for image encoding, the order from fastest to slowest was SP, SC, MTF, RP, GAF, and for the CNN models, it was VGGNet, ResNet, GoogleNet, DenseNet.

Table 4 displays the results of fault diagnosis accuracy based on the image encoding techniques and CNN models utilized. For the input data of the CNN model, considering the time continuity, the front dataset was employed sequentially for training (60%, 324 images), validation (20%, 108 images), and testing (20%, 108 images), and the accuracy was calculated accordingly. The experimental results showed that the fault diagnosis of the drive module used in this study achieved the highest performance with an accuracy of 96.0069% using the Spectrogram-based image encoding technique and DenseNet.

### 4.2. Fault Diagnosis Using CNN Based on Fault Data Generation and Image Encoding

**Figure 10:**
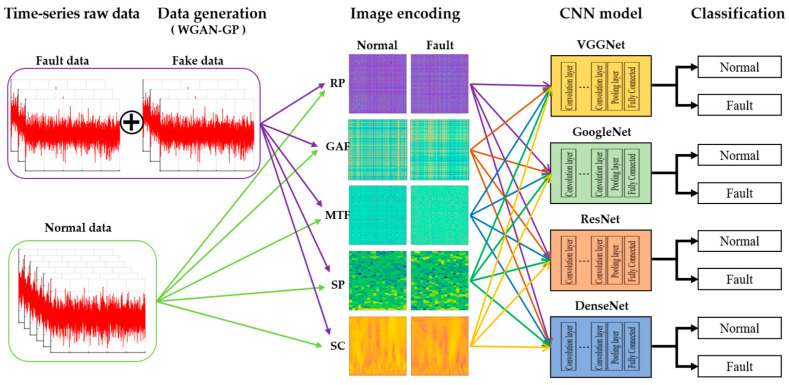
Method of fault diagnosis using CNN based on data generation and image encoding.

The final fault diagnosis procedure proposed in this paper is illustrated in Figure 10. The vibration data from the time-series were divided into normal and fault sections to extract data, and the insufficient fault data were additionally generated using the WGAN-GP model. Both normal and fault data were transformed into image data using five image encoding techniques, and these images were then subjected to four distinct CNN models to determine accuracy and assess performance.

The quantity of fabricated fault data points generated via the WGAN-GP model equaled 324, mirroring the number of fault data points employed in the training set delineated in Section 4.1. Consequently, a combined total of 648 fault data points (real 324 + fake 324) and an equivalent number of 648 normal data points (all real) were utilized for training. Furthermore, the identical dataset employed in Section 4.1 was applied for validation and testing, facilitating an effective evaluation of the model’s performance.

The computing times for fault diagnosis based on CNN using data generation and image encoding were as follows: The time taken to generate fault data with WGAN-GP was 3488.44 s (epoch: 20,000). The time taken to convert time-series data into 864 images (raw 540 + fake 324) through image encoding is as follows: RP took 314.71 s, GAF 358.17 s, MTF 317.2 s, SP 42.49 s, and SC 121.12 s. For the computing time for fault diagnosis using CNN models, based on Epoch 20, the times were: VGGNet 290.29 s, GoogleNet 327.36 s, ResNet 299.45 s, and DenseNet 560.87 s. The additional data generation through the WGAN-GP model is time-intensive due to its complexity. The total computing time for the implementation of the algorithm is presented in Table 5.

Table 6 exhibits the accuracy results of fault diagnosis based on CNN, utilizing data generation and image encoding. In comparison to the results in Table 4, where no fault data were generated, we observed that nearly all models demonstrated an improvement in fault diagnosis accuracy. On average, the performance of image encoding techniques ranked from best to worst as follows: SP, SC, GAF, MTF, RP; and for CNN models: Dense-Net, ResNet, GoogleNet, VGGNet. Ultimately, for the vibration dataset of the driving module used in this experiment, the fault diagnosis algorithm utilizing the SP image encoding technique and DenseNet CNN model achieved the highest performance with an accuracy of 99.8264%.

In this research, for the application of classification methods to the fault diagnosis model, the time-series data acquired from the durability tests of the driving module up to just before the failure were used. The normal section was set to the initial 50% of the dataset, and the fault section was defined as the 10 h period immediately preceding the failure. Future work will involve varying the proportions of the sections and conducting performance comparison experiments across multiple datasets to enhance the algorithm for more effective fault diagnosis.

## 5. Conclusions

In this paper, data generation models, image encoding techniques, and proven convolutional neural networks were utilized for the effective fault diagnosis of HCR used in industrial settings. The experiment extracted data for training by selecting the initial 50% of the lifespan as the normal section and the last 10 h prior to fault as the fault section, using time-series vibration data collected from durability tests of the drive module. Additionally, more fault data were generated using the WGAN-GP model. In this paper, considering that the performance of image encoding varies with different types of datasets, five image encoding techniques, RP, GAF, MTF, SP, and SC, were utilized to convert both normal and fault data into images. These images were then applied to four types of CNN models to calculate the diagnostic accuracy of each. The data generated from experiments were analyzed for similarity through statistical attribute comparison. Their performance was validated by application to the fault diagnosis model, confirming enhanced accuracy in post-generation fault diagnosis compared to pre-generation. It was established that the time-series vibration data from the driving module in this study exhibited the best performance with SP among the image encoding techniques and DenseNet among the CNN models, achieving a diagnostic accuracy of 99.8264%.

In upcoming work, rather than relying on a single dataset from a driving module, we will verify the performance of our algorithm using multiple datasets. We will also focus on comparing its performance with various existing fault diagnosis methods to ascertain the superiority of our algorithm. Additionally, we intend to procure data from HCR functioning in diverse industrial environments to develop a more robust fault diagnosis method. Consequently, the overarching objective of this research is to create fault diagnosis technology capable of monitoring operational robot statuses, detecting abnormalities before faults occur, and thereby contributing to improvements in industrial productivity and maintenance efficiency.

## Figures and Tables

**Table 1:** Driving conditions for the HCR module’s durability test.

Torque	Constant Speed	Acceleration/ Deceleration Speed	Acceleration/ Deceleration Time	Constant Speed Time	Stop Time	Cycle
115 N∙m	12 RPM	24 RPM/s^2^	0.5 s	4.5 s	1 s	13 s

**Table 2:** Similarity evaluation results of generated data.

Data Type	Evaluation Results of Generated Data (g)				
	Mean	Median	Standard Deviation (SD)	Skewness	Kurtosis
Normal data	0.1105	0.0924	0.0854	1.1301	1.6426
Fault data	0.2362	0.1974	0.1818	1.0818	1.2316
Generated data	0.2355	0.1981	0.1803	1.0786	1.3104

**Table 3:** Total computing time of fault diagnosis using image encoding and CNN.

Image	Computing Time for CNN Model-Based Fault Detection (s)			
	VGGNet	GoogleNet	ResNet	DenseNet
RP	366.06	401.39	378.58	556.28
GAF	390.56	425.89	403.08	580.78
MTF	365.63	400.96	378.15	555.85
SP	195.13	230.46	207.65	385.35
SC	245.55	280.88	258.07	435.77

**Table 4:** Results of fault diagnosis using image encoding-based CNN models.

Image	CNN Model-Based Fault Detection Accuracy (%)			
	VGGNet	GoogleNet	ResNet	DenseNet
RP	50.0000	67.2454	69.8495	69.5602
GAF	79.2245	86.9792	87.4421	87.8472
MTF	70.2546	70.7755	72.0486	69.5197
SP	94.6759	91.0880	95.5440	96.0069
SC	91.2616	92.5926	92.9977	88.9468

**Table 5:** Total computing time of fault diagnosis using CNN based on data generation and image encoding.

Image	CNN Model-Based Fault Detection Accuracy (%)			
	VGGNet	GoogleNet	ResNet	DenseNet
RP	4093.44	4130.51	4102.6	4364.02
GAF	4136.90	4173.97	4146.06	4407.48
MTF	4095.93	4133.00	4105.09	4366.51
SP	3821.22	3858.29	3830.38	4091.8
SC	3899.85	3936.92	3909.01	4170.43

**Table 6:** Results of fault diagnosis using data generation and image encoding-based CNN models.

Image	CNN Model-Based Fault Detection Accuracy (%)			
	VGGNet	GoogleNet	ResNet	DenseNet
RP	50.0000	95.0810	95.0810	96.4699
GAF	94.5023	96.7014	97.8009	96.6435
MTF	75.1157	94.3866	94.7338	97.0486
SP	99.4213	98.9583	99.4213	99.8264
SC	98.4954	98.5532	98.3796	98.4954

## Data Availability

The data used in the paper was collected through experiments supported by the project we conducted. Currently, it is difficult to disclose the data for additional experiments and research. In the future, we will strive to share our data by securing more data and conducting further research.
